# Diagnosis and treatment of MPN in real life: exploratory and retrospective chart review including 960 MPN patients diagnosed with ET or MF in Germany

**DOI:** 10.1007/s00432-023-04669-3

**Published:** 2023-03-08

**Authors:** Andreas Schmidt, Christiane Bernhardt, Dieter Bürkle, Stefan Fries, Carla V. Hannig, Kathleen Jentsch-Ullrich, Andreas Josting, Stephan Kreher, Marcel Reiser, Hans Tilman Steinmetz, Hans Tesch, Stephanie Terner, Alexander Schulte, Carl C. Crodel, Francesca Palandri, Florian H. Heidel

**Affiliations:** 1 Internal Medicine C, University Medicine Greifswald, Sauerbruchstrasse, 17475 Greifswald, Germany; 2 Gemeinschaftspraxis Hämatologie und Onkologie, Dortmund, Germany; 3 Zentrum für Ambulante Onkologie, Schorndorf, Germany; 4 Onkologische Schwerpunktpraxis, Bamberg, Germany; 5 Onkologische Praxis, Bottrop, Germany; 6 Gemeinschaftspraxis Hämatologie und Onkologie, Magdeburg, Germany; 7 OnkoBerlin, Berlin, Germany; 8 Hämatologisch-Onkologische Schwerpunktpraxis, Bad Liebenwerda, Germany; 9 Praxis Internistischer Onkologie und Hämatologie, Cologne, Germany; 10 MV-Zentrum für Hämatologie und Onkologie, Cologne, Germany; 11 Onkologie Bethanien, Frankfurt a.M., Germany; 12 Novartis Pharma GmbH, Nuremberg, Germany; 13 Department of Hematology and Oncology, University Hospital Jena, Jena, Germany; 14 IRCCS Azienda Ospedaliero-Universitaria di Bologna, Istituto di Ematologia “Seràgnoli”, Bologna, Italy

**Keywords:** Myelofibrosis, Essential thrombocythemia, MF, ET, Myeloproliferative neoplasia, MPN

## Abstract

**Purpose:**

The WHO 2016 re-classification of myeloproliferative neoplasms resulted in a separation of essential thrombocythemia (ET) from the pre-fibrotic and fibrotic (overt) phases of primary myelofibrosis (MF). This study reports on a chart review conducted to evaluate the real life approach regarding clinical characteristics, diagnostic assessment, risk stratification and treatment decisions for MPN patients classified as ET or MF after implementation of the WHO 2016 classification.

**Methods:**

In this retrospective chart review, 31 office-based hematologists/oncologists and primary care centers in Germany participated between April 2021 and May 2022. Physicians reported available data obtained from patient charts via paper–pencil based survey (secondary use of data). Patient features were evaluated using descriptive analysis, also including diagnostic assessment, therapeutic strategies and risk stratification.

**Results:**

Data of 960 MPN patients diagnosed with essential thrombocythemia (ET) (n = 495) or myelofibrosis (MF) (n = 465) after implementation of the revised 2016 WHO classification of myeloid neoplasms was collected from the patient charts. While they met at least one minor WHO-criteria for primary myelofibrosis, 39.8% of those diagnosed with ET did not have histological BM testing at diagnosis. 63.4% of patients who were classified as having MF, however, did not obtain an early prognostic risk assessment. More than 50% of MF patients showed characteristics consistent with the pre-fibrotic phase, which was emphasized by the frequent use of cytoreductive therapy. Hydroxyurea was the most frequently used cytoreductive medication in 84.7% of ET and 53.1% of MF patients. While both ET and MF cohorts showed cardiovascular risk factors in more than 2/3 of the cases, the use of platelet inhibitors or anticoagulants varied between 56.8% in ET and 38.1% in MF patients.

**Conclusions:**

Improved histopathologic diagnostics, dynamic risk stratification including genetic risk factors for cases of suspected ET and MF are recommended for precise risk assessment and therapeutic stratification according to WHO criteria.

## Introduction

Recommendations for diagnosis and treatment of patients with MPN have been recently revised by the WHO 2016, WHO 2022 and ICC 2022 classifications of myeloid neoplasms (Arber et al. 2016, 2022; Khoury et al. 2022). In 2016, this first revision resulted in re-classification of MPN patients’ diagnosis, specifically those previously diagnosed with essential thrombocythemia (ET) and myelofibrosis (MF). The introduction of a pre-fibrotic phase of primary myelofibrosis has changed the diagnostic landscape and raised the question of a potential need to re-classify patients’ diagnoses accordingly. This may have specific relevance for interpretation of clinical trials conducted prior to the WHO 2016 classification and compounds being approved for one of those MPN entities such as JAK-inhibitors for myelofibrosis. The majority of MPN patients in Germany receive their care in an outpatient (ambulatory) setting, despite the fact that the bulk of multicenter trials examining therapeutic methods for ET and MF have been established and carried out at specialized academic centers. Here, the approval of drugs for specific disease entities is highly relevant to make them available to patients without filing prior applications for re-imbursement. In this study, we sought to evaluate the treatment reality of patients diagnosed with ET or MF in real life after the 2016 update of the WHO classification in terms of clinical characteristics, diagnostic measures, use of risk assessment and therapy decisions. Data was analyzed from a chart review conducted at specialist practices for hematology and primary care centers.

## Patients and methods

### Objectives and aims of the study

This retrospective chart evaluation was performed in primary care settings with experience in treating patients with myeloproliferative neoplasms. The main objective of this investigation was to evaluate characteristics of patients with essential thrombocythemia (ET) and myelofibrosis (MF) in real life, especially in regard to diagnostic measures, the use of risk stratification, and therapy choices.

### Recruitment of participants

Participating centers have been identified through personal contact and email from a representative panel of board-certified hematologists in Germany. Centers that had successfully contributed to previous chart reviews (Crodel et al. 2022; Jentsch-Ullrich et al. 2016) were also included. As previously mentioned, doctors were required to devote more than 50% of their time to patient care. In total, 31 centers participated in this evaluation between April 2021 and May 2022. The chart review was performed as a paper–pencil based questionnaire and the documented data was compiled in an Excel-format. Participating centers received financial compensation for their contributions.

### Questionnaires and data acquisition

The identification of patients with the diagnosis of essential thrombocythemia (ET) or primary and secondary myelofibrosis (MF) has been conducted in an unbiased manner through the databases of each participating center. The questionnaire contained questions on (i) patient characteristics, (ii) medical history (iii) laboratory and molecular data, (iv) disease related symptoms, (v) medications and (vi) risk assessment. Following the completion of questionnaires, an investigator meeting of the 10 centers with highest recruitment numbers was conducted.

### Diagnosis, response criteria and risk scores

Patients with confirmed diagnosis of primary MF (PMF), secondary MF (post-PV MF, post-ET MF) or ET in 2016 or after according to institutional/local practice were included. For ET, only patients with at least one of the following characteristics were eligible: Anemia (Hb < 12 g/dl), splenomegaly, elevated LDH (> upper normal limit). The participants were instructed to use (i) CTCAE criteria for documentation of potential toxicities of the administered medications as well as (ii) ELN criteria to assess for indicators of progression (Barosi et al. 2010) and (iii) IWG-MRT-criteria (Tefferi et al. 2006) to assess for response.

### Ethics, patients’ consent and permissions

The results of the chart review were given as aggregate datasets for each center and no personally identifiable information was gathered. The institutional review board examined and approved the questionnaires and study materials.

## Results

### Patients’ characteristics

**Fig. 1:**
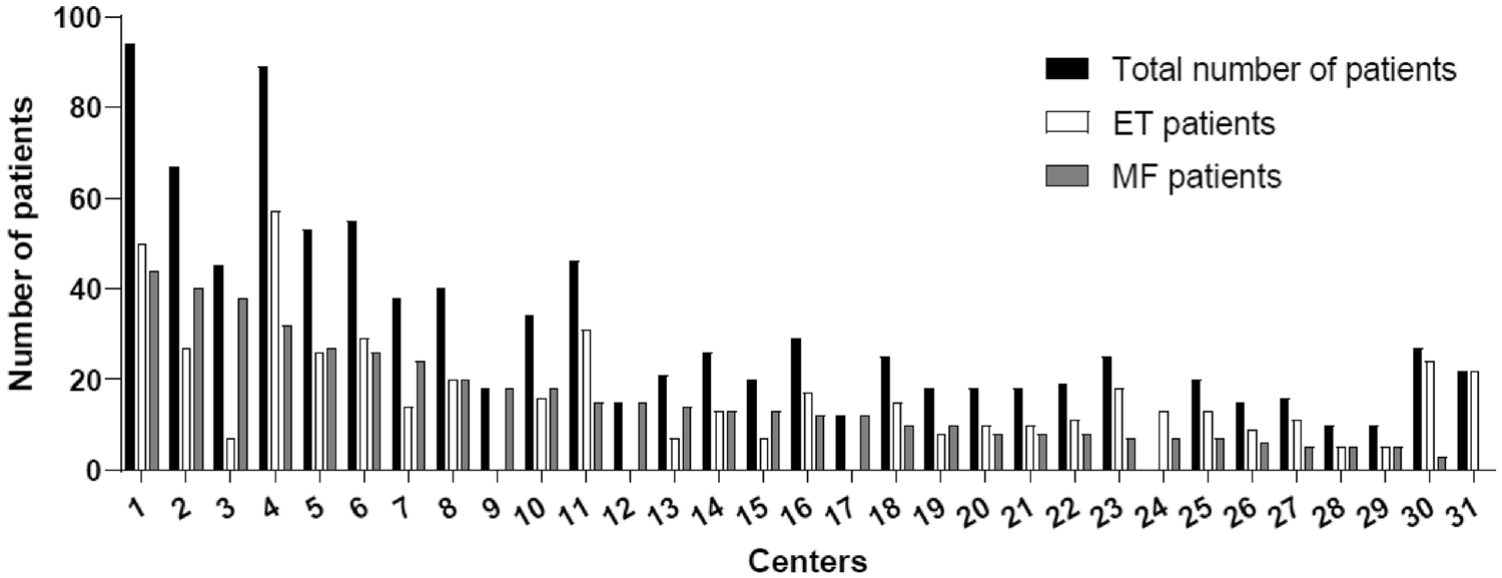
Participating centers and number of total patients (black bar), ET patients (white bar) and MF patients (grey bar) reported per center

In this analysis (data cut-off on May 31, 2022), a total of 960 patients diagnosed with essential thrombocythemia (n = 495) and myelofibrosis (n = 465) were reported by 31 centers (Fig. 1).

Patients received their diagnosis after publication of the 2016 WHO-classification of MPN. As anticipated, patients' gender distribution for ET (55.4% female vs. 44.6% male) was predominately female, whereas that for MF (50.5% female vs. 49.5% male) was balanced. The majority of MPN patients investigated here were of older age in both the ET- (63.4% > 65 years) and MF- (76.4% > 65 years) groups, respectively (Table 1).

**Table 1:** Patient characteristics

Characteristics	ET n = 495	MF n = 465
Biological sex		
Male	221 (44.6%)	230 (49.5%)
Female	274 (55.4%)	235 (50.5%)
Age		
< 50 years	68 (13.7%)	36 (7.7%)
< 50–60 years	92 (18.6%)	62 (13.3%)
> 60–70 years	104 (21.0%)	124 (26.7%)
> 70 years	228 (46.1%)	243 (52.3%)

Overall, the patient population was older than in previously published multicenter trials for ET or MF. As demanded by inclusion criteria, all patients in the primary analysis had been diagnosed after publication of the WHO 2016 re-classification of myeloproliferative neoplasms (Arber et al. 2016).

### Diagnostic assessment

**Fig. 2:**
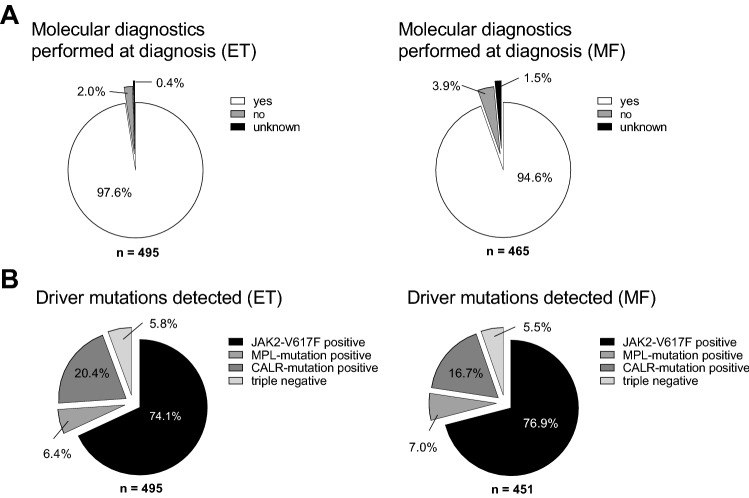
Molecular diagnostics and analysis of driver mutations in 495 patients classified as ET and 465 patients classified as MF. **A** Percentage of patients, who received molecular diagnostics at diagnosis. **B** Rate of driver mutations detected at diagnosis

Molecular diagnostics regarding detection of driver mutations was available in 97.6% of ET and 94.6% of MF patients (Fig. 2). *JAK2*V617F was detected in 74.1% (n = 337) ET and 76.9% (n = 320) of MF patients. In contrast, MPL-mutations were found in 6.4% (n = 29) and 7.0% (n = 29) and CALR-mutations in 22.2% (n = 101) and 18.7% (n = 78) patients with ET and MF, respectively. These findings show comparable rates of *JAK2, CALR* and *MPL*-mutations as recently published in a large dataset of 2035 MPN patients (Grinfeld et al. 2018), including patients with PV and MPN-U. Compared to molecular analyses of large ET and MF cohorts (Zoi and Cross 2017), this population shows a bias towards patients with *JAK2* mutations.

**Fig. 3:**
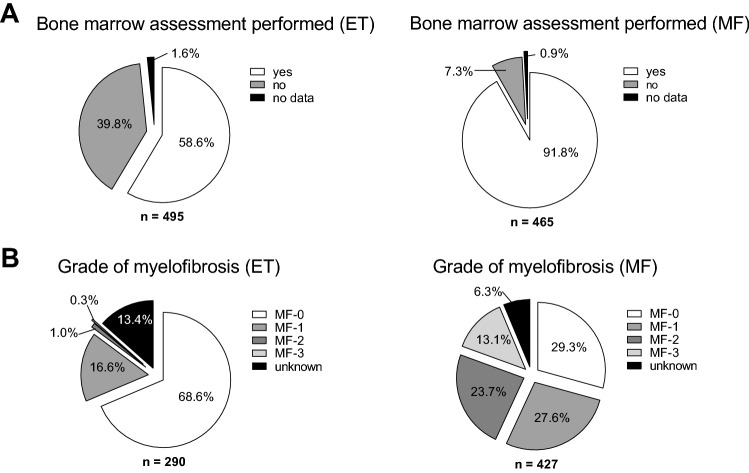
Bone marrow histology of 495 patients with ET and 465 patients with MF. **A** Bone marrow assessment at time of diagnosis. **B** Fibrosis grading at time of diagnosis, percentage of subgroup as indicated in **A**

Only 58.6% (n = 290) of patients diagnosed as ET had a bone marrow (BM)-biopsy performed as part of the examination at the time of initial diagnosis. (Fig. 3). In contrast, patients classified as MF had received BM histology in 91.8% of the cases (n = 427). Within the ET cohort, 85.2% (n = 247/290) showed myelofibrosis grade 0 or 1. Only 4/290 cases had a documented fibrosis grade of 2 or 3 that would not be compatible with the clinical diagnosis of ET. Of note, in 13.4% of patients (n = 39/290) the fibrosis grade was not available or documented in the histopathology report. In contrast, patients within the MF cohort were diagnosed with fibrosis grade 0 or 1 in 56.9% (n = 243/427) and with grade 2 or 3 in 36.8% (n = 157/427) of cases. Myelofibrosis grading was not available in 6.3% (n = 27/427) of patients classified as MF. Together, these findings show that across both cohorts 68.3% (n = 490) of patients receiving a bone marrow biopsy with available fibrosis grading had fibrosis grade 0 or 1. Thus, the majority of patients within the MF cohort fulfil histologic criteria of pre-fibrotic myelofibrosis according to the 2016 WHO classification.

Assessment of clinical characteristics focused specifically on parameters with relevance for diagnostic classification (ET versus MF) according to the WHO 2016 classification: spleen size assessment by palpation and ultrasound, lactate dehydrogenase levels (LDH), circulating blasts in the peripheral blood, white blood cell count (WBC) and hemoglobin levels (HGB) (Table 2).

**Table 2:** Clinical characteristics

Characteristics	ET n = 495	MF n = 465
Splenomegaly (> 11 cm)		
Yes	113 (22.8%)	272 (58.5%)
No	374 (75.6%)	188 (40.4%)
Palpable splenomegaly	5 (4.4%)	61 (22.4%)
No data (incl. not examined)	8 (1.6%)	5 (1.1%)
LDH		
Below upper normal	72 (14.5%)	45 (9.7%)
Above upper normal	398 (80.4%)	366 (78.7%)
No data	25 (5.0%)	54 (11.6%)
Blasts in peripheral blood		
≥ 1%	18 (3.6%)	36 (7.7%)
< 1%	251 (50.7%)	62 (13.3%)
No data	226 (45.7%)	124 (26.7%)
White blood cell count		
≥ 11.000/µl	134 (27.1%)	197 (42.4%)
< 11.000/µl	351 (70.9%)	250 (53.8%)
No data	10 (2.0%)	18 (3.8%)
Anemia		
Severe (Hb < 10 g/dl)	10 (2.0%)	65 (14.0%)
Mild (Hb ≥ 10 g/dl and < 12 g/dl)	62 (12.5%)	103 (22.2%)
No anemia (Hb > 12 g/dl)	414 (83.6%)	281 (60.4%)
No data	9 (1.8%)	16 (3.4%)
Platelet count		
≥ 450.000/µl	464 (93.7%)	290 (62.4%)
100.000–450.000/µl	22 (4.4%)	147 (31.6%)
< 100.000/µl	1 (0.2%)	9 (1.9%)
No data	8 (1.6%)	243 (52.3%)

Among the pre-selected ET patient cohort, 22.8% (n = 113) showed splenomegaly (> 11 cm diameter), and 1% (n = 5) by palpation; 80.4% (n = 398) had elevated LDH (above upper normal limit), 3.6% (n = 18) had circulating blasts, 27.1% (n = 134) elevated leukocyte values (> 11Gpt/l), 12.5% (n = 62) mild anemia (Hb ≥ 10 g/dl and < 12 g/dl) and 2% (n = 10) severe anemia (Hb < 10 g/dl). Without having had histological BM evaluation, the majority of patients who were categorized as ET showed at least one minor criterion indicative of pre-fibrotic MF. On the other hand, splenomegaly was more frequently identified in patients who were classified as MF: 58.5%; (n = 272) with > 11 cm diameters assessed by imaging and 22.4% (n = 61) by palpation. Moreover, patients classified as MF showed elevated peripheral blasts ≥ 1% (13.8%; n = 64), leukocytosis (42.4%; n = 197) or anemia (36.2%, n = 168; 14%, n = 65 with severe anemia Hb < 10 g/dl).

In summary, > 80% of the selected patient cohort classified as ET showed at least one diagnostic minor criterion for MF according to WHO 2016. More than 50% of patients classified as MF had findings consistent with pre-fibrotic disease. These findings indicate that the majority of patients from both ET and MF cohorts in this chart review, show criteria indicative for pre-fibrotic disease stage of myelofibrosis. However, clinical diagnosis of patients classified as ET could not be histologically confirmed due to lack of BM histology at diagnosis in 41.4% of patients.

### Symptom burden

MPN patients frequently report on disease-related symptoms that have negative impact on social interactions, productivity, physical activity, and quality of life (Harrison et al. 2017; Mesa et al. 2016). Symptom burden does not necessarily correlate with disease subtype, thromboembolic risk, or risk of disease progression. In this chart review analysis, classical constitutional symptoms such as fever, weight loss, night sweats general MPN-associated symptoms such as fatigue, abdominal pain, restricted physical mobility, cough, pruritus, skeletal pain, loss of appetite, and other symptoms were assessed as reported at initial diagnosis.

**Fig. 4:**
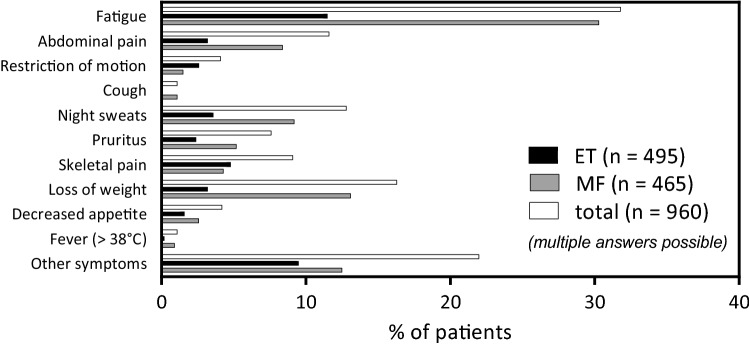
Percent of patients reporting on specific MPN-associated symptoms at time of diagnosis (multiple answers possible)

At time of diagnosis, the classical constitutional symptoms fever, night sweats and weight loss were reported in 0.7, 13.0 and 11.6% of patients in the ET cohort and 1.7, 18.0 and 25.5% of patients in the MF cohort, respectively (Fig. 4). Besides constitutional symptoms, fatigue was the most prominent symptom documented for both ET (41.3%) and MF (59.0%), followed by abdominal pain (ET 11.6%; MF 16.3%), skeletal pain (ET 17.4%; MF 8.4%), pruritus (ET 8.7%; MF 10.0%), restriction of motion (ET 9.4%; MF 2.9%), decreased appetite (ET 5.8%; MF 5.0%), and cough (ET 0.0%; MF 2.1%).

Overall, the reported symptom burden appeared to be more prominent in MF than in ET patients, except for skeletal pain. Of note, the symptom burden documented in the patients’ charts appeared less when compared to symptoms published previously in either clinical trials (Harrison et al. 2012; Verstovsek et al. 2010) or patient-reported questionnaires (Harrison et al. 2017; Mesa et al. 2016). These findings are consistent with differences reported between physician- and patient-reported symptoms in one of our recent reports (Jentsch-Ullrich et al. 2016).

### Risk stratification

35.3% (n = 164) of patients had their MF risk classification scored at the time of diagnosis. Most frequently used scoring systems included static scoring systems such as IPSS (37.8%, n = 62) which is validated at the timepoint of diagnosis and dynamic scoring systems such as DIPSS (24.4%, n = 40), DIPSS-plus (16.5%, n = 27) for primary myelofibrosis (PMF) and MYSEC-PM (11.6%, n = 19) for secondary myelofibrosis (SMF). Interestingly, molecular scores were used in less than 8% of cases. Overall, 25% of MF patients presented as low-risk, 42.1% as intermediate-1 risk, 21.3% as intermediate-2 risk and 11.6% as high-risk at the timepoint of diagnosis. To assess whether structured risk assessment was more frequently performed at later timepoints, the questionnaire asked for prognostic scoring in the further course of the disease. Unexpectedly, a prognosis score assessment was only performed in 12.9% (n = 60) of MF patients at later time points. Here, dynamic and molecular/genetic scoring systems were more frequently used compared to the scores used at primary diagnosis: 30% (n = 18) DIPSS-plus, 3.3% (n = 2) MIPSS70, 21.7% (n = 13) MIPSS70plus 2.0 and 30% (n = 18) MYSEC-PM. Of note, IPSS was still used in 10% of patients, although not formally validated for dynamic assessment. A higher proportion of MF patients (50%, n = 30) were categorized as int-2 or high risk. These numbers may indicate selection of a high-risk subgroup with early clonal progression or secondary myelofibrosis.

### Therapeutic strategies

**Fig. 5:**
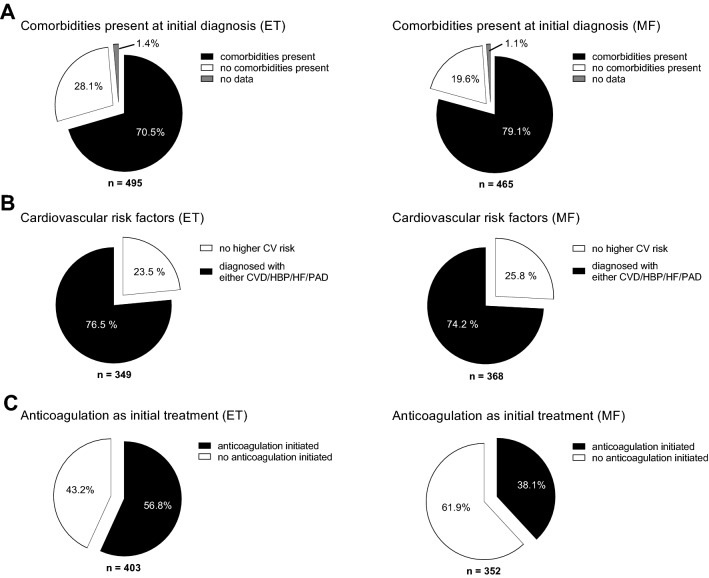
Prevalence of cardiovascular risk factors and anticoagulation. **A** Prevalence of general comorbidities at time of diagnosis. **B** Prevalence of cardiovascular risk factors prevalent at time of diagnosis, percentage indicated as percent of patients with comorbidities (as indicated in **A**). **C** Anticoagulation (incl. platelet inhibitors, heparins, DOACs, OACs) initiated at time of diagnosis, percentage based on number of patients, who received a pharmacological therapy at time of diagnosis (403 ET and 352 MF)

Thromboembolic (TE) complications are a clinical challenge in patients with ET but also pre-fibrotic myelofibrosis. Therefore, we aimed to assess for cardiovascular risk, use of anticoagulants and disease-specific therapeutic strategies (Fig. 5).

**Fig. 6:**
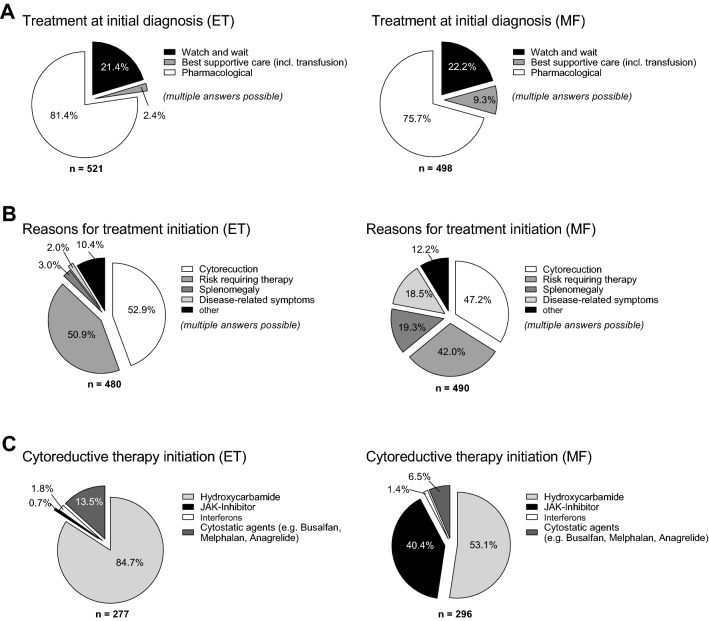
Pharmacologic therapy in 495 patients with ET and 465 patients with MF. **A** Treatment initiated at time of diagnosis (multiple answers possible), **B** Reason for initiation of pharmacotherapy (multiple answers possible). **C** Pharmacological cytoreduction chosen at time of diagnosis, percentage of patients as indicated in **B**

70.5% (n = 349/495) ET patients and 79.1% (n = 368/465) MF patients reported on comorbidities at diagnosis. 76.5% (n = 267/349) of ET patients and 74.2% (n = 273/368) of MF patients had cardiovascular comorbidities defined as cardiovascular disease (CVD), arterial hypertension (AH), heart failure (HF) or peripheral artery disease (PAD). Anticoagulation including platelet inhibitors (ASA or P2Y inhibitors), heparins, direct oral anticoagulants (DOACs) and oral anticoagulants (OACs) as part of the initial therapy following diagnosis was documented for 56.8% (n = 229/403) of ET and 38.1% (n = 134/352) MF patients. Watchful waiting was the primary treatment choice for 21.4% of ET (n = 106) and 22.2% of MF (n = 103) patients. 2.4% (n = 12) of ET and 8.4% (n = 39) of MF patients were transfusion dependent, respectively (Fig. 6). After diagnosis, 84.7% (n = 233) of ET patients received hydroxycarbamide (HC) for pharmacologic cytoreduction. 56.8% (n = 229) were treated with anticoagulants.

Similar to this, 38.1% (n = 134) of MF patients received anticoagulant treatment, and 53.1% (n = 155) received HC medication after diagnosis. At diagnosis, 62.4% (n = 290) of MF patients had platelet counts that were high (> 450 Gpt/l). JAK inhibitors were administered to 40.4% (n = 118) of the patients receiving symptom-focused therapy. For both ET and MF (> 40% of patients), cytoreduction and TE risk-reduction were the primary justifications for starting medication. In 19.3 and 18.5% of the cases, respectively, splenomegaly and symptom management were further justifications for MF treatment. Taken together, pharmacologic therapy was focused on cytoreduction which is consistent with the high numbers of ET and pre-MF cases in this cohort. Anticoagulants were used less frequently in patients classified as MF despite the high number of patients presenting as pre-fibrotic MF.

## Discussion

The WHO 2016 classification has integrated multiple parameters such as clinical aspects, molecular and genetic data as well as histomorphology to diagnose myeloproliferative neoplasms (Passamonti and Maffioli 2016). Among other aspects, this development led to the definition of a pre- fibrotic phase of primary myelofibrosis (pre-PMF). Pre-PMF can be distinguished from ET by bone marrow morphology, has a higher rate of disease progression to the fibrotic phase of myelofibrosis or acute leukemia, and is characterized by inferior survival. In clinical practice, this development made several adjustments necessary: (i) stringent evaluation of bone marrow histology, (ii) re-evaluation of clinical diagnoses and risk assessment performed before 2016 as well as (iii) information of patients regarding a potential change in classification of their disease. Data on these adjustments made necessary by an update of the WHO classification in real life are rare, specifically in countries such as Germany, where the majority of MPN patients are treated in private practices (by office-based hematologists) outside of academic centers. In this chart review analysis, we investigated diagnostic procedures, clinical characteristics, risk assessment and treatment decisions of MPN patients diagnosed after publication of the WHO 2016 classification, either with MF or with ET despite presenting with at least one minor criterion for myelofibrosis (anemia, blasts, splenomegaly, elevated LDH or leukocytosis).

Of note, the proportion of *JAK2* mutated patients was significantly higher in this selected cohort compared to published molecular studies (Zoi and Cross 2017). The presence of *JAK2* driver mutations is associated with higher systemic inflammation (Perner et al. 2019), thromboembolic risk (Barbui et al. 2012) and disease progression (Tefferi et al. 2018).

Despite the presence of clinical parameters potentially indicative for (pre-fibrotic) myelofibrosis, only 58.6% of ET patients received histopathologic assessment of their bone marrow at diagnosis. Discussion of these results with the participating centers and investigators revealed lack of approval by the patients for a bone marrow biopsy in the majority of cases and focus on quality of life for the longest possible duration. In contrast, approval was more frequently obtained at later time points, when signs of disease progression became evident. This may result in further diagnostic blur of this analysis, as distinction between primary and secondary myelofibrosis is hardly possible without prior histopathologic assessment. Diagnostic procedures are conducted differently in academic centers and other European countries with clear recommendations for initial histologic BM assessment. Of note, BM morphology is critical, and its analysis requires expert pathologist assessment. Critically, consensus among experts in the distinction between ET and pre-PMF ranges from 53 to 88% (Passamonti and Maffioli 2016). This heterogeneity emphasizes the importance of inter-professional discussion of histologic assessment and integration of clinical with histopathologic data by hematologists. Moreover, precision of MPN diagnoses also relies on exclusion of reactive and inflammatory conditions. Classification of MPN subtypes is not only critical to inform patients about risk of disease progression and probability of survival but also regarding access to approved pharmacologic therapies. As shown in this chart review, more than 10% of patients classified as ET or MF report on significant symptom burden and may benefit from JAK-inhibitor treatment, approved for the treatment of disease-related splenomegaly or symptoms in adult patients with myelofibrosis. Conversely, the need for cytoreductive therapies to achieve TE risk reduction has been perceived and cytoreductive treatment had been initiated according to guideline recommendations (mainly using hydroxycarbamide/hydroxyurea). This is of major importance considering the large number of patients in this chart review showing clinical and histopathologic findings consistent with pre-fibrotic myelofibrosis.

In contrast, risk assessment regarding disease progression of myelofibrosis has been conducted in only 35.3% of cases. This finding is of critical importance as 47.7% of MF patients documented in this study were below 70 years of age and therefore potentially eligible for allogeneic stem cell transplantation as a curative approach. Moreover, scoring systems that include genetic information were used in less than 50% of patients while scores including molecular information (driver mutations, high risk mutation) were used in less than 10% of cases at diagnosis. Especially for transplant-eligible patients, the use of established molecular risk scores (such as MIPSS70; (Guglielmelli et al. 2018)) is recommended to identify younger patients with high risk for disease progression and curative potential (Griesshammer et al. 2021). However, among the limitations of this chart review is the nature of aggregated datasets: allocation of risk score assessment to specific individual patients (e.g. those with eligibility for stem cell transplantation or clinical risk signs) cannot be analzyed.

Together, while meeting minimal criteria for primary myelofibrosis, more than 40% of individuals categorized as ET did not receive histological BM testing at diagnosis. On the other hand, early prognostic risk assessments were not given to more than 60% of individuals who were classified as having MF. In conclusion, improved histopathology assessment and dynamic risk stratification including genetic risk factors for cases of suspected ET and MF are recommended for precise risk assessment and therapeutic stratification according to WHO criteria.

## Data Availability

Materials described in the manuscript, including all relevant raw data, will be freely available to any researcher wishing to use them for non-commercial purposes, without breaching participant confidentiality.
